# Pregnancy outcomes in people with spinal cord injury: A cross-sectional study

**DOI:** 10.1177/1753495X261435108

**Published:** 2026-03-26

**Authors:** Anne Berndl, Georgia Condran, Claire Mazzia, Anne Harris

**Affiliations:** 1 Temerty Faculty of Medicine, 7938 University of Toronto, Toronto, Ontario, Canada; 2 Accessible Care Pregnancy Clinic, Division of Maternal-Fetal Medicine, Department of Obstetrics and Gynecology, 71545 Sunnybrook Health Sciences Centre, Toronto, Ontario, Canada; 3 School of Occupational and Public Health, 7984 Toronto Metropolitan University, Toronto, Ontario, Canada; 4 Dalla Lana School of Public Health, University of Toronto, Toronto, Ontario, Canada

**Keywords:** Pregnancy, spinal cord injury, disabled persons

## Abstract

**Background:**

There is limited information surrounding the obstetric and neonatal outcomes of people with spinal cord injury (SCI).

**Methodology:**

This cross-sectional study is a subgroup analysis of an international survey examining reproductive and urogenital health in people with SCI. Key obstetric and neonatal outcomes were assessed using descriptive analysis.

**Results:**

A total of 216 participants from 33 countries were observed; of the reported 262 pregnancies ending in live or stillbirth, bladder infections occurred in 80 (30.5%), kidney infections in 15 (5.7%), hospital admission in 44 (16.8%) and ICU admission in 6 (2.3%). Preterm birth occurred in 68 of 262 pregnancies (25.9%) and neonatal intensive care unit admission occurred in 38 of 257 pregnancies (14.8%).

**Conclusion:**

People with SCI appear to be at risk of perinatal complications including bladder and kidney infections and hospital admission. This information is of value to people with SCI who are contemplating pregnancy or are currently pregnant and their healthcare providers.

## Background

Spinal cord injury (SCI) is a common cause of disability globally, involving damage to the spinal cord from trauma, disease or degeneration that affects function. Spinal cord injury remains a significant public health concern, often resulting in lifelong disability and impacts to motor and sensory function, emotional health and quality of life.^1–3^ Overall well-being in people with SCI may be impacted by physical symptoms (e.g., spasticity, pain and autonomic dysreflexia), level of community support and bodily autonomy.^4^

People assigned female at birth make up 20% of those with SCI.^5^ Fertility is typically preserved in people with SCI, allowing many to consider pregnancy.^6,7^ One prior study found that 14% of individuals with SCI became pregnant after their injury.^8^ Despite this knowledge, the intersection between SCI and pregnancy remains a limited area of research, raising important questions about maternal and fetal health outcomes, and potential effects to pre-existing medical conditions.^9,10^

Currently available literature is comprised of smaller scale investigations with potential for significant bias, revealing a significant knowledge gap in our understanding of the unique challenges for pregnant people with SCI.^6^ Some studies suggest potential risks, including increased rates of preterm birth (PTB) and caesarean section, preterm rupture of membranes and exacerbation of pre-existing medical conditions including increased rates of urinary tract infections (UTIs).^9,11,12^ Currently available studies have demonstrated that urologic concerns including UTIs and pyelonephritis appear to be the most common complication during pregnancy and reason for perinatal hospitalization in people with SCI.^9,13^ Recurrent UTIs may be related to an increased risk of abortion, prematurity and low birth weight.^14^ Other studies have demonstrated that pregnancy outcomes can be positive with appropriate preconception counselling and multidisciplinary obstetric care.^15^

However, comprehensive data remains scarce, leading to uncertainty among healthcare providers (HCPs) about best practices for management of pregnant people with SCI. As sexual and reproductive health outcomes are critical considerations for people with SCI contemplating pregnancy, there is a pressing need to synthesize data analyzing pregnancy and SCI, specifically related to obstetric outcomes, physical health changes and quality of life in this population.

This study aims to assess our current understanding of pregnancy in the context of SCI, to highlight questions that remain unanswered and provide key recommendations for the care of this population. This knowledge may contribute to the development of targeted clinical guidelines including preconception counselling and anticipatory interventions to enhance the care offered to people with SCI during their reproductive years.

## Methodology

This study is a sub-analysis of an international observational questionnaire examining reproductive and urogenital health outcomes in people with SCI. The questionnaire was developed with principles of person-centred research in collaboration with people with SCI. This cross-sectional survey obtained research ethics approval through Sunnybrook Health Sciences (REB: 085-2019). Recruitment techniques, including snowball and peer recruitment, were used to distribute the online questionnaire in four languages through social media and SCI organizations.

To be included in this sub-analysis, individuals needed to be over the age of 18, be assigned female at birth and have a self-reported diagnosis of SCI, and experience a pregnancy.

One hundred seventy one people had at a total of 262 pregnancies that resulted in live or stillbirth. These 171 individuals responded to optional questions about experiences during their pregnancy. This study assessed demographic data, and key obstetric and neonatal outcomes in this population. Descriptive analysis was completed for each pregnancy.

## Results

Results are from 33 countries; recruitment rate was 85.4% (1056/1237) and completion rate was 73.8% (780/1056). Of these, 216 people reported a total of 392 pregnancies, 5 of which were twin pregnancies; 171 people had at a total of 262 pregnancies that resulted in live or stillbirth. The average parity among included individuals was 2.5. In addition, the mean body mass index (BMI) of the included pregnancies was 23.14. Demographic data of the eligible 171 individuals is presented in Table 1.

**Table 1.** Characteristics of the study sample of 171 people who had a live or still birth.

Variable	Participants (*n* = 171)
Mean age at SCI (years)	18.8 ± 7.7
Mean maternal age (years)	28.8 ± 5.5
Country of residence	*N* (%)
United States	104 (61.8)
Canada	23 (13.5)
Australia and New Zealand	18 (10.5)
United Kingdom and Ireland	10 (5.8)
Spain	7 (4.1)
Other	9 (5.3)
Level of injury	
Cervical	58 (33.9)
Thoracic	88 (51.5)
Lumbar	22 (12.9)
Sacral	3 (1.8)
Severity of injury	
ASIA-A	65 (38.0)
ASIA-B	39 (22.8)
ASIA-C	30 (17.5)
ASIA-D	33 (19.3)
ASIA-E	3 (1.8)
Unreported	1 (0.6)

The mean age at SCI was 18.8 ± 7.7 years and the mean maternal age was 28.8 ± 5.5 years. The majority of individuals were from the United States (104, 61.8%). The most commonly reported level of SCI was cervical (58, 33.9%) and severity of SCI was ASIA-A (65, 38.0%).

As shown in Table 2, 257 (68.5%) pregnancies resulted in livebirth and 5 (1.3%) in stillbirth. The majority of pregnancies were delivered vaginally (160, 61.1%).

**Table 2.** Pregnancy outcomes.

Variable	Pregnancies
Pregnancy outcomes of 392 pregnancies	*N* (%)
Livebirth	257 (68.5)
Stillbirth	5 (1.3)
Miscarriage	71 (18.9)
Termination	39 (10.4)
Ectopic	3 (0.8)
Currently pregnant	17 (4.5)
Mode of delivery of 262 live and stillbirths	
Vaginal delivery	160 (61.1)
Caesarean delivery	98 (37.4)
Unreported	4 (1.15)

**Table 3.** Maternal antenatal complications of 262 live or stillbirths.

Variable	Pregnancies (*n* = 262); *N* (%)
Gestational diabetes	12 (4.5)
Impaired glucose tolerance	3 (1.1)
Hyperemesis	17 (6.5)
Sleep apnea	2 (0.7)
Pneumonia	1 (0.3)
High blood pressure	16 (6.1)
Pre-eclampsia	5 (1.9)
Antenatal bleeding	9 (3.4)
Anaemia	43 (16.4)
Peripartum cardiomyopathy	0 (0.0)
Cardiac arrhythmia	2 (0.7)
Acute fatty liver of pregnancy	0 (0.0)
Cholestasis	3 (1.1)
PUPPs	1 (0.3)
Renal failure	0 (0.0)
Bladder infection	80 (30.5)
Kidney infection	15 (5.7)
DVT or PE	2 (0.7)
Stroke	2 (0.7)
Admission to hospital	44 (16.8)
Admission to ICU	6 (2.3)
Falls	13 (4.9)

Maternal complications are summarized in Table 3. Bladder infections occurred in 80 (30.5%) and kidney infections occurred in 15 (5.7%) of 262 pregnancies ending in live or stillbirth. There were no pregnancies with reported renal failure.

Pre-eclampsia occurred in 5 (1.9%) of 262 pregnancies. Deep vein thrombosis (DVT) or pulmonary embolism (PE) occurred in 2 (0.7%) and stroke occurred in 2 (0.7%) pregnancies; 44 (16.8%) pregnancies were complicated by admission to hospital and 6 (2.3%) were complicated by ICU admission.

Postpartum complications are summarized in Table 4. The most commonly reported outcomes included postpartum depression (PPD; 30, 11.5%), baby blues (30, 11.5%), bladder infection (24, 9.5%), autonomic dysreflexia (17, 6.5%) and pain (30, 11.5%). Deep vein thrombosis occurred in 3 (1.1%) of people in the postpartum period; 3 (1.1%) people experienced caesarean wound infections.

**Table 4.** Postpartum complications of 262 live or stillbirths.

Variable	Pregnancies (*n* = 262); *N* (%)
Endometritis	4 (1.5)
Autonomic dysreflexia	17 (6.5)
Retained POC	3 (1.1)
Postpartum haemorrhage	9 (3.4)
Postpartum depression	30 (11.5)
Postpartum blues	30 (11.5)
DVT	3 (1.1)
Stroke	0 (0.0)
Pre-eclampsia	1 (0.3)
C-section wound infection	3 (3.1)
Bladder infection	24 (9.5)
Pain	30 (11.5)
Other	24 (9.1)

Table 5 summarizes neonatal outcomes. Of 262 pregnancies, 68 (25.9%) resulted in PTB. Birth weight data of 260 live or stillbirths is included. Birth weights were between the 10^th^ and 90^th^ percentile in 47.7% of pregnancies, with birth weights less than or equal to the 10^th^ percentile in 20.4% of pregnancies.

**Table 5.** Neonatal outcomes.

Gestational age of 262 live or stillbirths.	
Variable	Pregnancies (*n* = 262); *N* (%)
≥37 weeks (term)	194 (72.9)
<37 weeks	68 (25.9)
≤34 weeks	31 (11.8)
≤30 weeks	12 (4.5)
GA of stillbirths (weeks)	20, 20, 20, 25, 38
Birth weight percentage of 260 live or stillbirths.	
Variable	Pregnancies (*n* = 260)
≥95^th^ percentile	14 (5.4)
≥90^th^ percentile	21 (8.1)
10^th^–90^th^ percentile (AGA)	124 (47.7)
≤10^th^ percentile	53 (20.4)
≤5^th^ percentile	28 (10.8)
≤2.5^th^ percentile	20 (7.7)
Complications of 257 livebirths.	
Variable	Pregnancies (*n* = 257)
NICU admission	38 (14.8)
Jaundice	57 (22.2)
Breathing problems	24 (9.3)
Feeding problems	20 (7.8)
Medical complications	20 (7.8)

In terms of neonatal complications, neonatal intensive care unit (NICU) admission occurred in 38 pregnancies (14.8%). Other reported complications included jaundice, breathing problems and feeding problems.

## Discussion

More people with disabilities, including SCI, are choosing to become pregnant.^7^ However, there remains limited data on the management of SCI in pregnancy. The current study aimed to utilize a large international survey about SCI to address the paucity of research evaluating obstetric and neonatal outcomes of people with SCI. This analysis supports a previously reported concern for a risk of PTB in this population, SGA, as well as high incidence of bladder and kidney infections, and hospital admission.^10,12^

In terms of maternal antenatal complications, the present study found that antenatal pre-eclampsia occurred in 5 (1.9%) and postpartum pre-eclampsia in 1 (0.3%) pregnancy. These rates are similar to baseline risk in the general population. One systematic review found that pre-eclampsia complicates approximately 4.6% of all deliveries.^16^

The present study also found increased incidences of critical, and rare, outcomes in the antenatal period. Venous thromboembolism (VTE), including DVT or PE occurred in 2 (0.7%) pregnancies antenatally. This observed risk is consistent with trends in current research, which highlight immobility in pregnancy as a risk factor for VTE.^17^ Okoroh et al., explained how pregnant people face 4–5 times increased VTE risk when compared to non-pregnant people.^17^ These risks may be exacerbated in people with physical disabilities, including SCI, who face pre-existing challenges related to mobility.^18^ In addition, 3 (1.1%) people experienced DVT in the postpartum period. People with SCI are at an increased baseline risk for DVT, although research supports the highest risk is in the first year after SCI and then risk decreases thereafter.^19^ Current data recommends risk assessment during the antepartum and postpartum period to tailor mechanical prophylaxis and anticoagulation strategies in people with physical disabilities during pregnancy.^20^ There remains a need for guidance on best practices for VTE prophylaxis in people with physical disabilities.

Stroke occurred in 2 (0.7%) pregnancies. The first person was 18 years old at the time of their SCI, and they used a power wheelchair to aid with mobility. They had a BMI of 19.5 and a history of hypotension and hypothyroidism. They were 20 years old at the time of pregnancy. Their pregnancy was unexpected and was complicated by pre-eclampsia and an emergency caesarean delivery due to autonomic dysreflexia. The second person was 32 years old at the time of their SCI, and they used a manual wheelchair to aid with mobility. They had a BMI of 30.9 and a history of hypercholesteremia and thromboembolism (designated by survey wording as ‘blood clots in legs/lungs’). They were 32 years old at the time of pregnancy. Their pregnancy was unexpected, and they delivered vaginally following induction. They experienced autonomic dysreflexia during pushing. While both individuals who experienced a stroke had a history of autonomic dysreflexia, it cannot be determined whether this was causal of stroke. Stroke is a serious and rare antenatal complication. One meta-analysis estimates that stroke complicates 30.0 per 100,000 pregnancies.^21^ Numerous studies to date have found an increased incidence of VTE in pregnant people with disabilities, which may explain the observed increase risk of stroke in this population, although additional investigations are required.^18^

Consistent with previous research, the present study found that individuals with SCI experienced an increased risk of UTIs (30.5%) and acute pyelonephritis (5.7%) (designated by survey wording as ‘kidney infections’) during pregnancy.^10,12,22^ Studies have shown that UTIs are prevalent among pregnant people with SCIs, with one study reporting rates of 48%^23^ and another reporting rates of urinary complications as high as 59%.^6^ Sterling et al. reported recurrent UTIs occurring in 32% of individuals and neurogenic bladder in 53%.^15^ Neurogenic bladder dysfunction, another common complication in this population, may be related to increased vulnerability to UTIs in pregnancy. The common practice of intermittent catheterization in individuals with SCIs further contributes to the increased risk of UTIs.^12^ These complications may predispose pregnant people with SCI to increased rates of hospitalization as observed in the present study (16.8%).

The finding of increased incidence of preterm labour among pregnant individuals with SCI (25.9%) is also aligned with previous research.^6,22^ One study reported an incidence of 18%, which was increased to 25% in people with higher level lesions.^9,11^ Ghidini et al. found that PTB occurred in 33% of individuals, with 22% unable to feel preterm labour.^6^ The underlying mechanism of this association has yet to be elucidated, but may be related to autonomic dysreflexia and infections (UTIs, intrauterine infections).^10^ These complications may be amendable to preventative interventions including heightened surveillance and appropriate prophylaxis.^6^ Importantly, the present study included 12 (4.5%) pregnancies with a gestational age at delivery <30 weeks. There is significant morbidity associated with deliveries <34 weeks. This finding supports the notion that people with SCI can have complicated courses of labour and delivery and require evidence-based anticipatory care.^10^

Previous research has suggested that UTIs are a common pregnancy complication which increase the risk of preterm labour in the general obstetric population.^24^ Further, pyelonephritis has been identified as a risk factor for low birth weight, PTB and fetal death.^25^ The increased incidence of infections in people with SCI could contribute to these complications.^15^ However, these links have not been sufficiently investigated in the context of pregnant people with SCI.

The current study also identified an increased risk of neonates being born less than tenth percentile (20.4%). Importantly, this study was able to assess the birth percentiles of neonates and found that 10.8% were less than the 5^th^ percentile and 7.7% were less than 2.5^th^ percentile. This is key information, as neonates with smaller birth weight percentiles have more adverse outcomes.^26^ This is consistent with previous research which showed that people with SCI tend to have more complicated courses of labour and delivery and low birth weight infants.^10^ Significant differences observed in neonatal head circumference may imply further consideration be given to the impact of SCI on fetal growth and uteroplacental insufficiency. The risk of NICU admission was also increased for infants born to individuals with SCI (14.8%), which may be related to the increased risk of PTB and its associated complications. One study examining neonatal outcomes among people with disabilities, including SCI, reported that neonates born to people with disabilities had a higher risk of NICU admission, with an adjusted relative risk of 1.70 compared to those without disabilities^27^

In terms of postpartum complications, PPD was experienced after 30 (11.5%) of the included pregnancies. This is similar to PPD baseline risk, which is estimated to be 10–15% in developed countries.^28^ Current literature suggests that people with physical disabilities are at higher risk of PPD when compared to those without disabilities, although more research is needed.^29^ In addition, 3 (3.1%) pregnancies were complicated by caesarean section wound infection postpartum. This is similar to baseline risk in the general population. A Canadian study found that surgical site infections following caesarean delivery occurred in 2.7% included participants.^30^ Of the three reported caesarean section wound infections, one person reported using a wheelchair to aid with mobility. This is an important consideration for people who use wheelchairs who are contemplating elective caesarean section.

The present study found a low prevalence of individuals with sleep apnea (2, 0.7%). Previous research reports increased rates of sleep apnea among people with SCI compared to the general population.^31^ It is known that pregnancy is a risk factor for sleep apnea, which may contribute to rates of SGA seen in the present study. However, this is an area for further investigation.

This study is subject to limitations inherent to survey-based research, including the reliance on self-reported data and the cross-sectional nature of the study which may have impacted results.

Given limitations inherent to survey-based research, Table 6 compares the incidence of adverse pregnancy outcomes reported in previous studies. Analysis in the described studies is completed by pregnancy. There is variation in definition of adverse pregnancy outcomes among studies. Comparison is further complicated by variability in degree and severity of SCI among study participants. The present study remains the largest to date examining pregnancy outcomes among people with SCI. Further, the recruitment methods may have been less likely to capture the experiences of people least and most affected by their injury as they may be less active in SCI groups. Approximately 95% of individuals were living resource-rich countries at the time of survey completion. Another limitation is that the survey had non-mandatory questions and analysis was conducted on questions with responses. As well, the results could be strengthened with an assessment of risk based on level and degree of SCI, which could be beneficial in providing more personalized counselling and care. The strengths of this study lie in the inclusion of detailed demographics of a large sample of people with SCI.

**Table 6.** Comparison of adverse pregnancy outcomes in previous studies.

Variable	Present study	Ghidini et al., 2008	Robertson et al., 2020	Crane et al., 2019
Study methodology	International survey	US-based survey	UK retrospective observational study	US retrospective cohort study (relative risk compared to controls)
Population	257 livebirths in 168 people	37 livebirths in 24 people	66 livebirths in 50 people	161 livebirths in 161 people
Pregnancy outcomes				
Preterm delivery (<37 weeks)	21.0%	33%	18%	RR 1.31
Low birth weight (<2500 g)	18.7%	16%		
Small for gestational age (<10%ile)	20.4%		10%	RR 1.32
Hypertensive complications		14%	4.5%	
Pre-eclampsia/eclampsia	1.9%			RR 1.29
Thrombotic events	0.8%	8%	4.5%	
Autonomic dysreflexia	7.8%	27%	6%	
Urinary tract infections/pyelonephritis	30.7%	51%	24%	RR 14.9
Gestational diabetes	4.7%			RR 0.92
Stroke	0.8%			

## Conclusion

This study supports previously reported concerns for a risk of PTB, and bladder and kidney infections in people with SCI, as well as high incidence of low-birth-weight infants and NICU admission. In addition, the present study identified an increased risk of rare, critical antepartum complications including VTE and stroke. Insights gained from this study may be of benefit to people with SCI who are contemplating pregnancy or are currently pregnant, and the interdisciplinary providers including obstetricians, ICU physicians, physiatrists, nurses, physiotherapists and neurosurgeons who care for them. Results may be used to inform HCPs of perinatal complications observed in pregnancies among people with SCIs and best practices for antenatal counselling and proactive management of pregnancy complications. Although the majority of pregnancies resulted in term, vaginal deliveries, this study suggests a need for increased surveillance during pregnancy for fetal growth and PTB, consideration for risk of thromboembolic disease and need for thromboprophylaxis as well as development of screening and treating protocols for UTI. Future research into the causes of and ways to prevent fetal growth restriction, PTB and urologic infection in people with SCI, as well as possible links between these outcomes is needed.
